# 

**Published:** 1915-02-20

**Authors:** 


### Personalia.

Mr. William Fry, the Dublin solicitor who took an active part in the conversion of Dublin Castle into a hospital, received the honour of knighthood from Lord Aberdeen before the Viceroy's farewell reception at Dublin Castle last Saturday.

The position of Chief Clerk at the Hampstead General and North-West London Hospital has been filled by the appointment of Mr. A. J. Parker, formerly of the Samaritan Hospital and the East London Hospital for Children.

### Medical Appointments.

Dr. E. H. Hicks, one of the two medical representatives of the Board of Governors of the Leicester Royal Infirmary representing the Leicestershire and Rutland Branch of the British Medical Association, has been appointed by the British Red Cross Society to the Molassis Hospital, near Boulogne (typhoid fever). Dr. Hicks graduated B.A. Camb. in 1880, and qualified L.S.A. in 1886, and M.R.C.S. Eng. a year later. He has had considerable experience of medical practice abroad, having been medical officer to the British and United States Legations at Bagola, South America. He has contributed several papers on leprosy to the medical journals.

**RATES OF SUBSCRIPTION: Twelve months, 6/6; Six months, 4/-; Three months, 2/-, post free to any address in the United Kingdom. Abroad, Twelve months, 8/8; Six months, 5/-; Three months, 2/6, post free.—Address The Subscription Dept., "THE HOSPITAL," The Hospital Building, 28/29 Southampton Street, Strand, London, W.C.**

